# Design and Performance of a J Band MEMS Switch

**DOI:** 10.3390/mi10070467

**Published:** 2019-07-13

**Authors:** Naibo Zhang, Ze Yan, Ruiliang Song, Chunting Wang, Qiuquan Guo, Jun Yang

**Affiliations:** 1 The 54th Research Institute of China Electronic Science and Technology Group Corporation, Beijing 100070, China; 2 Beihang University, Beijing 100083, China; 3 Department of Mechanical & Materials Engineering, University of Western Ontario, London, ON N6A 3K7, Canada

**Keywords:** J band, MEMS, switch

## Abstract

This paper presents a novel J band (220–325 GHz) MEMS switch design. The equivalent circuits, the major parameters, capacitance, inductance and resistance in the circuit were extracted and calculated quantitatively to carry out the radio frequency analysis. In addition, the mechanical property of the switch structure is analyzed, and the switching voltage is obtained. With the designed parameters, the MEMS switch is fabricated. The measurement results are in good agreement with simulation results, and the switch is actuated under a voltage of ~30 V. More importantly, the switch has achieved a low insertion loss of ~1.2 dB at 220 GHz and <~4 dB from 220 GHz to 270 GHz in the “UP” state, and isolation of ~16 dB from 220 GHz to 320 GHz in the “DOWN” state. Such switch shows great potential in the integration for terahertz components.

## 1. Introduction

There is a growing interest in terahertz components in the frequency from 100 GHz to 10 THz for potential applications in security scanning, atmospheric monitoring, medical imaging and ultrafast wireless communications. Silicon-based terahertz monolithic integrated circuits for communication circuits and radar systems have been developed with high integration and low cost as the main advantages [1]. With the growing requirement for multi-function and reconfigurability of THz circuits and systems, the tunable devices have become necessary for research, in which switches are the most basic components. At the same time, the switch is also the key component to control the high frequency signal in the communication system [1,2,3]. However, semiconductor switches are not suitable for terahertz frequency applications due to insertion loss, power handling capacity and linearity limitations.

RF micro-electromechanical (MEMS) switches have demonstrated excellent linearity, high isolation, low insertion loss and low power consumption [3]. Previous studies on MEMS switches have focused on frequencies lower than 100 GHz and have achieved good performances [4,5]. In comparison, RF-MEMS switches can be integrated with coplanar waveguide (CPW) transmission lines and together with other RF devices. In recent research, THz communication systems are already being developed. To meet this requirement of terahertz system, a wideband RF-MEMS switch using BiCMOS process was firstly presented in [1]. It realized a return loss of 24 to 12 dB and an insertion loss of 1.2 to 2.7 dB within the frequency range 180−250 GHz. After that, other RF-MEMS switches fabricated on chip were also proposed [2,3]. On the other hand, MEMS-based waveguide switches for terahertz band were demonstrated in [6,7]. Most recently, RF-MEMS waveguide switch has achieved an isolation of 19–24 dB and insertion loss of 2.5–3 dB for 500–750 GHz, respectively. In a recent publication, the authors have studied these MEMS switches in the frequency band between 500 and 750 GHz [3,6,7], which was the highest frequency RF MEMS device reported so far, with an insertion loss of 2–4 dB and DC driving voltage of 30–60 V.

This paper introduces a novel J Band (220 GHz–325 GHz) MEMS switch with operating frequency from 220 GHz to 320 GHz. The equivalent circuits are discussed and extracted for the switch, and the parameters in the circuit are calculated quantitatively. The measurement results show a good agreement with simulation results, and the insertion loss is ~−1.2 dB in the “UP” state when the frequency is 220 GHz, and the isolation is better than 16 dB across the band in the “DOWN” state. The switch is actuated under a voltage of ~30 V.

## 2. Design

**Figure 1:**
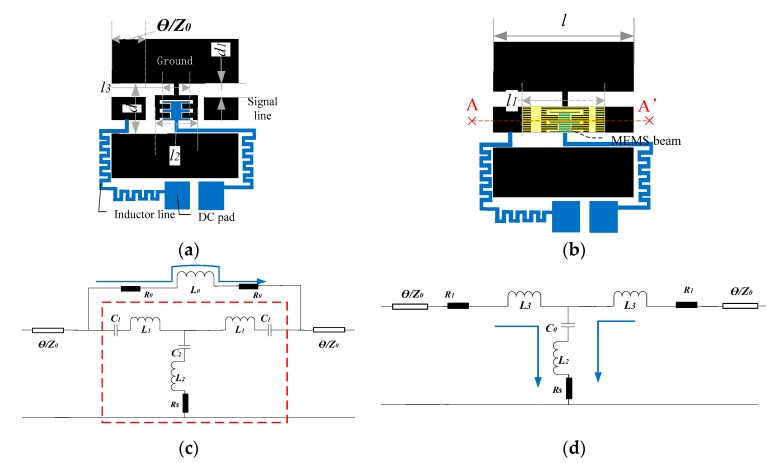
THz RF micro-electromechanical (MEMS) switch. (**a**) Switch structure without beam, *d* = 60 μm, *d*_1_ = 18 μm, *l*_2_ = 60 μm, *l*_3_ = 40 μm. (**b**) Switch structure with beam, *l* = 204 μm, *l*_1_ = 134 μm. (**c**) The equivalent circuit of structure when the switch is in the “UP” state. (**d**) The equivalent circuit of structure when the switch is in the “DOWN” state.

The MEMS switch is designed as Figure 1a,b; the switch consisted of a CPW (coplanar waveguide) transmission line, DC bias and switch beam. By controlling the DC bias voltage, the switch beam can be brought into contact with the CPW section to switch into the “DOWN” state. To realize a switch working at THz frequencies, there are three major challenges: low insertion loss in the “UP” state, high isolation in the “DOWN” state, and robust mechanical properties. Reducing the capacitance of the switch to the ground can reduce the insertion loss of the switch in the “UP” state, so the ground structure between signal line is designed as the comb-like structure in Figure 1a. A driving voltage electrode plate is designed to complement the ground comb, which will increase the inductance value and thus increase the isolation of the signal to the ground. MEMS switch beam directly connecting the signal lines can make the switch inductance smaller in the “UP” state, which can reduce the reflected signal energy. In order to improve the isolation in the “DOWN” state, the capacitance value in the “DOWN” state needs to be increased, which is realized by covering a thin isolation layer Si_3_N_4_ on the bottom plate of the switch with a thickness of ~0.1 μm.

### 2.1. Radio Frequency Analysis

The equivalent circuits are analyzed in order to achieve a low insertion loss in the “UP” state and a high isolation in the “DOWN” state. The equivalent circuits of structure in the “UP” and “DOWN” states are shown in Figure 1c,d. The inductors *L*_0_, *R*_0/1_ are generated by MEMS switch beam, *C*_1_ is the capacitor produced by the gap between signal line and ground, *L*_1_, *L*_2_ are produced by ground between two signal line, and *C_2_* is the capacitor between switch beam and ground.

From the equivalent circuits, we can determine that there are three parameters to be quantitatively calculated: resistance (*R*), inductance (*L*) and capacitance (*C*). The inductance and capacitance can be calculated by formulas in literatures [8], and the resistance [9] is calculated as following.

**Figure 2:**
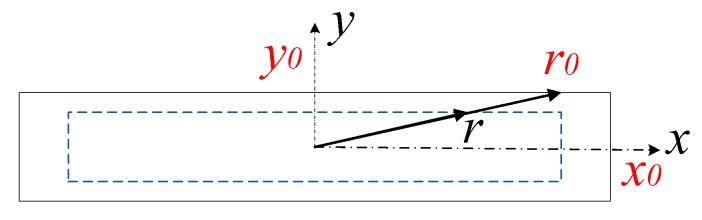
Side view of current distribution in metal film.

Current distribution is not uniform when the device is working at high frequencies due to skin effect in metal film; a diagram of the current distribution profile is illustrated in Figure 2. Due to the skin effect of current, the current mainly distributes from *r* to *r*_0_, and the current density is expressed as,

(1) $$J=J_{0}e^{-\alpha (r_{0}-r)},\;\alpha =\sqrt{\frac{w\mu \sigma }{2}}$$

where, *J*_0_ is surface current density, *α* is attenuation constant, *r*_0_ is the outer length of current distribution, *r* is the inner length of current distribution, *w* is frequency, *σ* is conductivity (= 4.1 × 10^7^ S/m), *μ* is magnetic permeability, and the skin depth of current is given as,

(2) $$\Delta =\sqrt{\frac{2}{w\mu \sigma }}$$

The current consists of the current density integral of the whole plane,

(3) $$I=\iint J_{0}(e^{-\alpha (x_{0}-x)}+e^{-\alpha (y_{0}-y)})dS\approx \frac{1}{\sqrt{2}}\pi \sigma E_{0}\frac{1}{\alpha }\sqrt{x_{0}{}^{2}+y_{0}{}^{2}}=\frac{1}{\sqrt{2}}\pi \sigma E_{0}\Delta \sqrt{x_{0}{}^{2}+y_{0}{}^{2}}$$

where, *x*_0_ and *y*_0_ are border lengths, *E*_0_ is the equivalent electrostatic field.

(4) $$R=\frac{E_{0}l}{I}=\frac{l\sqrt{2}}{\pi \sigma \Delta \sqrt{x_{0}{}^{2}+y_{0}{}^{2}}}=\frac{1}{2\times 10^{4}\times \sqrt{x_{0}{}^{2}+y_{0}{}^{2}}}$$

where, *l* is unit length, the parameters are designed as, *x*_0_ = 12 μm, and *y*_0_ = 0.5 μm.

In order to obtain good radio frequency performances, including low insertion loss in the “UP” state and high isolation in the “DOWN” state, it can be obtained by calculation that when the switch is in the “UP” state, its equivalent circuit parameters are: *R*_0_ = 2.4 Ω, *L*_0_ = 24 pH, *C*_1_ = 6.7 fF, *L*_1_ = 19 pH, *C*_2_ = 5 fF, *L*_2_ = 6.5 pH, and *R*_s_ = 2.5 Ω, respectively. When the switch is in the “DOWN” state, the equivalent circuit parameters are: *R*_1_ = 2.4 Ω, *L*_3_ = 23 pH, *C*_0_ = 134 fF, *L*_2_ = 6.3 pH, and *R*_s_ = 2.5 Ω, respectively.

The parameters of the switch structure can be calculated from the capacitance, inductance and resistance calculation formulas, where the capacitance and inductance calculation formulas obtained from the formula in literature [8], and the resistance is calculated from Formula (4). According to the equivalent circuit, calculated parameters and optimization, the parameters of the MEMS switch can be obtained as follows. The switch dimensions in Figure 1 are as, *Z*_0_ = 50 Ω, *d* = 60 μm, *d*_1_ = 18 μm, *l*_2_ = 60 μm, *l*_3_ = 40 μm, *l* = 204 μm, and *l*_1_ = 134 μm, respectively.

**Figure 3:**
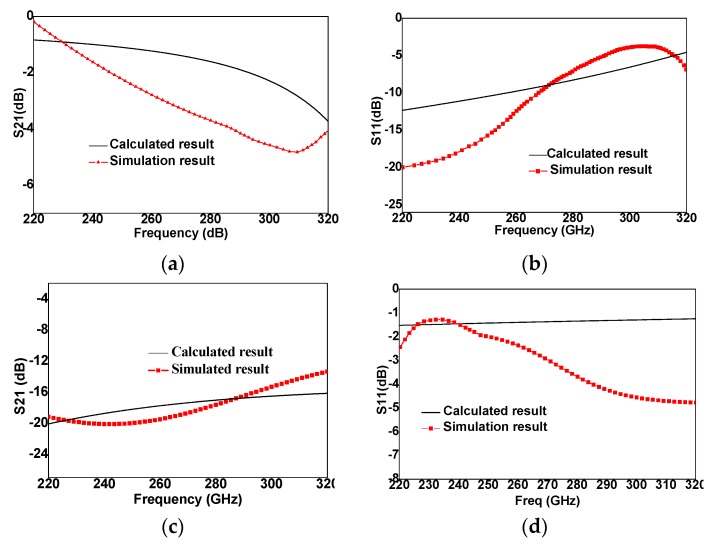
Calculated circuit and simulated S-parameters of MEMS switch. (**a**) Insertion loss in the “UP” state. (**b**) Return loss in the “UP” state. (**c**) Isolation in the “DOWN” state. (**d**) Return loss in the “DOWN” state.

The calculated results based on calculation parameters are compared with those of switch structure simulated based on finite element method, which are shown in Figure 3, and the frequency range is from 220 GHz to 320 GHz. When the MEMS switch is in the “UP” state, the insertion loss is ~−2.5 dB, and both two curves of simulation and calculation results have the same trend of change. When the MEMS switch is in the “DOWN” state, the isolation is ~16 dB, while two curves have some differences. The main reason for the deviation between two curves in Figure 3a,b is that the capacitance and inductance produced by the comb structure of the bottom electrode in the switch are small, which are neglected in the equivalent circuit, while these parameters are all considered in the simulation of the equivalent circuit in terahertz band. The difference in Figure 3d is because the capacitance and inductance between signal line and ground is ignored, which leads to ~2 dB differences when frequency is from 280 GHz to 320 GHz. The MEMS switch structures simulations are carried out by Ansoft HFSS, and circuits simulations are carried out by ADS.

### 2.2. Analysis of Mechanical Properties

**Figure 4:**
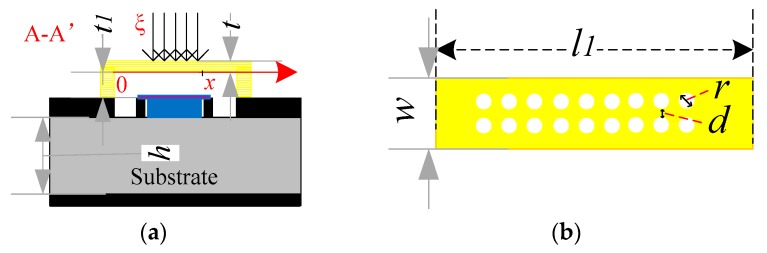
(**a**) The side view of switch, *t* = 1 μm, *t*_1_ = 1 μm, *t*_2_ = 1 μm. (**b**) The MEMS switch beam.

The MEMS switch is a symmetrical structure. Figure 4a shows the side view of the switch, the electrode plate is from (*l*_1_ − *x)* to *x*, and the electrostatic force is loaded in the middle part of the beam, with the whole size of the beam as *l*_1_. *ξ* is the pressure. The elastic coefficient (*k*) of MEMS switch is in two parts, *k*_1_ and *k*_2_.

(a) The elastic coefficient *k*_1_ is due to the stiffness of the bridge, which accounts for the material characteristics such as Young’s modulus [10], *E* (Pa), and the moment of inertia, *I* (m^4^*)* = *l*_1_*t*^2^*/*12, *t* is the thickness of switch beam.
  The deflection (*y*_1_) found by evaluating the integral is,
  (5) $$y_{1}=\frac{1}{EI}\int_{l_{1}-x}^{x}\frac{\xi }{48}(l_{1}{}^{3}-6l_{1}{}^{2}a+9l_{1}a^{2}-4a^{3})da$$
  Elastic coefficient (*k*_1_) caused by Stiffness of Beams is,
  (6) $$k_{1}=-\frac{\xi l_{1}}{y_{1}}=32Ew{\left(\frac{t}{l_{1}}\right)}^{3}\frac{1}{8{\left(x/l_{1}\right)}^{3}-20{\left(x/l_{1}\right)}^{2}+14(x/l_{1})-1}$$

(b) The elastic coefficient *k*_2_ is due to the biaxial residual stress, *S*’(Pa) = *σ*(1 − *ν*)*l*_1_*t*, where *ν* is the Poisson’s ratio [8].
  The deflection (*y*_2_) found by evaluating the integral is,
  (7) $$y_{2}=-\int_{l_{1}-x}^{x}\frac{\xi }{2S}(l_{1}-a)da$$
  Elastic coefficients induced by biaxial residual stresses is,
  (8) $$k_{2}=-\frac{\xi l_{1}}{y_{2}}=8\sigma (1-\nu )w(\frac{t}{l_{1}})\frac{1}{3-2(x/l_{1})}$$

Figure 4b is the MEMS structure beam, and holes on the beam surface are designed. Photoresist was used as sacrificial material and removed to fabricate the through holes. The hole diameter *r* = 5 μm, and the distance *d* = 8 μm; eighteen holes are designed in two rows on the beam surface. The holes will bring certain elastic coefficient error which has been well characterized in the literature [11]. The holes are helpful to release some of the residual stress in the beam but will lead to the reducing of the Young’s modulus [12]. The reduction of the residual stress is equivalent to *σ* = (1 − *μ*)*σ*_0_, where *σ*_0_ is the residual stress with no holes. The elastic coefficient is revised to *k* = *λ*_1_*(k*_1_ + *k*_2_*)*, (0 < *λ*_1_ < 1).

The total elastic coefficient is,

(9) $$k=\lambda (k_{1}+k_{2})=32Ew{\left(\frac{h_{3}}{l_{1}}\right)}^{3}\frac{\lambda }{8{\left(x/l_{1}\right)}^{3}-20{\left(x/l_{1}\right)}^{2}+14(x/l_{1})-1}+8\sigma (1-\nu )w(\frac{h_{3}}{l_{1}})\frac{\lambda }{3-2(x/l_{1})}$$

Through analysis and calculation, the parameters in this equation *λ*, *σ*, *E*, *t*, and *k* are 0.75, 56 MPa [13], 46 GPa [13], 1 μm, and 5.9 N/m, respectively. The controlled DC voltage is represented as *V* = *t*_1_[2*k*·Δ*d/*(*ε*·*ε_0_*·S)]^0.5^ [8], *ε*_0_ = 8.85 × 10^12^ F/m, *ε* = 1, Δ*d* is the displacement of switch beam, *S* is the area of electrodes, and *k* is the elastic coefficient. The calculated DC voltage is ~35 V.

## 3. Results

**Figure 5:**
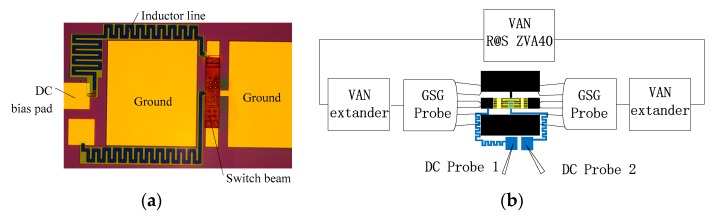
(**a**) Fabricated THz MEMS switch. (**b**) Set up of the two-port on wafer measurement.

In order to verify the switch design, the switches were fabricated, and the performances were measured. An image of the completed MEMS switch is provided in Figure 5a, and a two-port on wafer measurement is set up as shown in Figure 5b. R&S ZVA 40 vector network analyzer and VNA extender were used for testing the *S* (*S*21, *S*11) parameters, and the CASCADE probe station was used for measurement. All experiments were performed under ambient environment without any packaging. A G-S-G probe with 100 μm spacing was used for MEMS switch testing, and a through-reflect-line (TRL) calibration was applied using WinCal XE calibration software. The “Line” standards are used for higher calibration accuracy [3]. Calibrated measurement shows the loss of the 50 Ω CPW is around ~1.8 dB/mm at 200 GHz and around ~2.5 dB/mm at 300 GHz, which is consistent with the simulated results.

**Figure 6:**
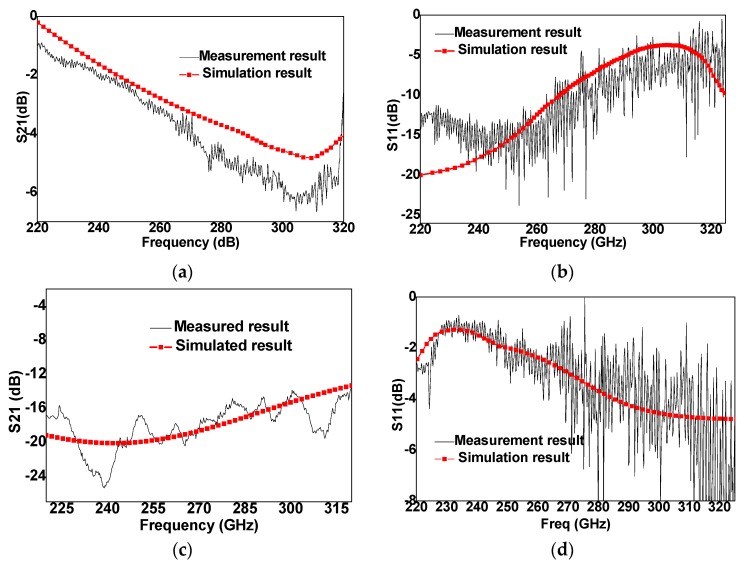
Measured and simulated results. (**a**) Insertion loss at “UP” state. (**b**) Return loss at “UP” state. (**c**) Isolation at “DOWN” state. (**d**) Return loss at “DOWN” state.

Figure 6 shows the RF performance of MEMS switch, and the frequency is from 220 GHz to 320 GHz. The black color curves are simulation results and red color curves are experimental results. The experimental results show good agreement with simulation results. When MEMS switch is in the “UP” state, the insertion loss is ~−1.2 dB at 220 GHz, and is <−4 dB from 220 GHz to 270 GHz. When MEMS switch is in the “DOWN” state, the isolation is ~<−16 dB from 220 GHz to 320 GHz. The DC voltage is ~30 V, which is less than calculated result (35 V), the reason is that the surface of switch beam is reduced and the hole on the switch is enlarged, which directly leads to the decrease of the elasticity coefficient of the switch, thus, the driving voltage of the switch being reduced. In addition, the out-of-plane deformation of a cantilever beam with residual stress gradient will also reduce the driving voltage [14,15].

## 4. Discussion

When the MEMS structure have been determined, the insertion loss is mainly influenced by two parts, (1) loss caused by the substrate dielectric constant and the substrate thickness, which is ~200 μm in this study. In order to reduce the insertion loss, the thickness of the substrate can be further reduced, such as 50 μm; The influence of the parameters of the switch on the switch RF performance is analyzed in two aspects: Firstly, circuit and theoretical analysis. Secondly, optimization simulation by simulation software. In this paper, related work has been undertaken in the early stage. The structure parameters of the switch are obtained through the analysis of the above two aspects. In the design, the thickness of the substrate is not the optimal value, but it is limited by the previous process, so the thickness of the substrate has not been reduced. (2) Loss caused by the fabrication process; there are two factors, fabrication tolerance and surface roughness. The deviation of the structure size often leads to impedance mismatch, which the surface roughness produced by fabrication limit often leads to increasing loss. So, in addition to the structural design, the loss caused by the thickness of the substrate and the loss caused by the fabrication process should be considered for the practical application use. Almost 10 samples were tested, and the results were almost the same. A discrepancy between simulation and measurement results is observed; a large fluctuation exists in the experimental data of Figure 6c, and a low agreement appears in Figure 6b (low frequency) and Figure 6d (high frequency). The reasons are as follows: (1) The parasitic parameters, such as inductance and capacitance, produced by the smoothness of the switch film surface will affect the RF performance parameters of the switch. (2) Ansof HFSS is used to simulate the RF performance of the switch. The size of the peripheral air box is set at the designing, but no metal package is added during the test, which will also affect the RF performance, resulting in some inconsistencies between the simulation and test results [16,17]. Furthermore, the main reasons for the uncertainty information about the experimental results is as follows: (1) Uncertainties from fabrication. Fabrication errors have great impact on RF performance, mainly due to the height of anchor zone and film thickness. The height of switch gap (anchor height) and film thickness designed here are 1 μm. When the height of switch gap changes, the RF performance of switch will be greatly affected. In addition, the size of the hole has an impact on the performance. When the diameter of the hole is less than (3–4)*t*_1_ and the switch is in the “UP” state, the influence of the hole is neglected. This is because the edge effect makes the hole “filled”, but when it is in “DOWN” state, the capacitance will be affected, where the influence ratio is the proportion of the film area reduced. At the same time, the fabrication will bring over-corrosion effect, the hole diameter will be larger than the designed diameter, which will affect the “UP” capacitor and further affect the radio frequency performance. (2) Uncertainties in testing, such as test instruments, test operation, etc. During testing, the contact between probe and switches will affect the test results. When the contact is insufficient, the contact resistance and inductance will increase, resulting in deviation of test results, such as resonance frequency offset. In addition, when the test transmission line interface is not fully connected or the transmission line jitters, there will be an impact on the test results.

To compare the proposed THz MEMS switch in this study and reported THz switch, Table 1 shows recently published THz MEMS switches. The proposed switch with a low insertion loss which is comparable to the switch performances [1,2,6], and low DC driving voltage [1,2,3,6,7]. In other words, the proposed switch can have both the advantages of a low DC driving voltage, a low insertion loss and high return loss of passband. Compared with the switches with the same center frequency (220 GHz) in literature [1], the switch in this study shows a much better performances than performances in literature [1]. When compared with literatures [3,6,7], the proposed switch in this study shows a lower DC driving voltage. These advantage features of proposed switch will be useful for many system applications, such as, MEMS dives and THz communication system.

## 5. Conclusions

A novel J band MEMS switch is designed, analyzed, fabricated, and measured. A low insertion loss in the “UP” state and high isolation in the “DOWN” state were achieved. The equivalent circuits retaining all the components were used and the switch parameters in the circuit were calculated quantitatively. The MEMS switch was fabricated, and the completed device was actuated under a voltage of ~30 V. The experimental results matched expectation and fit well with the simulation results. A switch with the insertion loss of −1.2 dB in “UP” state when the frequency is 220 GHz, and isolation better than 16 dB across the band in the “DOWN” state was fabricated.

## Figures and Tables

**Table 1:** Comparison of different THz switches.

Title 1	Frequency (GHz)	S21 (dB)	S11 (dB)	DC Driving Voltage (V)
Ref. [1]	170–220	1.9 dB@220 GHz	−12	50
Ref. [2]	110–170	−1.5	−19	60
Ref. [3]	500–750	2.7 dB@600 GHz	−15	60
Ref. [6]	330–500	−5	−20	95
Ref. [7]	500–750	−3.5	−16	36
This work	220–3200	1.2 dB@220 GHz	−16	30
